# Predictive Ability of Procalcitonin for Acute Kidney Injury: A Narrative Review Focusing on the Interference of Infection

**DOI:** 10.3390/ijms22136903

**Published:** 2021-06-27

**Authors:** Wei-Chih Kan, Ya-Ting Huang, Vin-Cent Wu, Chih-Chung Shiao

**Affiliations:** 1 Department of Nephrology, Department of Internal Medicine, Chi-Mei Medical Center, Yongkang District, Tainan 710, Taiwan; rockiekan@ntu.edu.tw; 2 Department of Biological Science and Technology, Chung Hwa University of Medical Technology, Rende District, Tainan 717, Taiwan; 3 Department of Nursing, Camillian Saint Mary’s Hospital Luodong, Yilan 265, Taiwan; frankie7451@gmail.com; 4 Division of Nephrology, Department of Internal Medicine, National Taiwan University Hospital, Taipei 100, Taiwan; q91421028@ntu.edu.tw; 5 Division of Nephrology, Department of Internal Medicine, Camillian Saint Mary’s Hospital Luodong, Ylan 265, Taiwan; 6 Saint Mary’s Junior College of Medicine, Nursing and Management, Yilan 266, Taiwan

**Keywords:** acute kidney injury, infection, predictor, procalcitonin, sepsis

## Abstract

Acute kidney injury (AKI) is a common yet complicated clinical entity with high morbidity and mortality. An essential strategy to improve AKI patients’ prognoses is finding optimal biomarkers to identify AKI in a timely manner. Procalcitonin (PCT), a well-recognized biomarker for diagnosing infection and guiding antibiotics therapy, has been proposed to predict AKI development and recovery in many clinical settings. The current review provides comprehensive and updated information from relevant studies to evaluate PCT’s AKI-predictive ability and the influence of infection on this predictive ability. PCT has demonstrated optimal predictive ability for AKI in various populations irrespective of infection. However, the predictive ability seems to be blunted by infection since infection and inflammation have a more potent influence than AKI on PCT elevation. We furthermore explain the complicated association between elevated PCT levels and AKI in infection and inflammation situations and recommend directions for further investigations to clarify the essential issue. In conclusion, although conflicting data exist, serum PCT level is a potential biomarker for predicting AKI in many clinical settings regardless of infection. Nevertheless, further studies are warranted to clarify the association between PCT, infection, and AKI and to confirm the utilization of PCT for AKI prediction.

## 1. Introduction

Acute kidney injury (AKI) is a common yet complicated clinical entity that comprises heterogeneous mechanisms and carries increased mortality and morbidity [1,2]. In recent decades, the improvement in AKI therapies and AKI patients’ prognoses have been limited [3,4]. An essential strategy for resolving the disappointing situation is finding promising biomarkers that could detect the development, etiology, location, type, and severity of kidney injury in a timely manner [5,6].

Procalcitonin (PCT) is a precursor of calcitonin with 116 amino acids and a molecular weight of 13,600 Da. Under a non-inflammatory situation in healthy individuals, many factors such as elevated calcium, glucagon, glucocorticoid, or calcitonin gene-related peptide would stimulate calcitonin production in thyroid C cells. This process enhances CALC-1 gene expression, which subsequently increases calcitonin mRNA, PCT, and calcitonin production in thyroid C cells. However, when individuals are in an inflammation or infection situation, calcitonin production is independent of the above regulations [7] and is alternatively stimulated by two mechanisms. These mechanisms contain direct and indirect pathways, which take place in many other organs, including the brain, heart, lung, liver, kidney, pancreas, and small intestine [7]. Since PCT is primarily formed and converted to calcitonin within the thyroid C cells, a low serum PCT level (0.05 ng/mL) exists in healthy subjects [7]. In cases of sepsis, PCT levels vary between 10 and 100 ng/mL, along with elevated cytokines including interleukin-1, interleukin-6, and tumor necrosis factor-α [8]. PCT is eliminated through the kidneys and liver [9]. In the infection or inflammatory state, PCT rises rapidly within the first 3–4 h of the event’s onset, peaks in 6–12 h, and decreases after 24 h until a normalization within five days [10]. PCT has been well recognized as an optimal biomarker for identifying infection and sepsis [11] and for guiding antibiotics therapy in critically ill patients with severe infection [12]. In addition to being a biomarker for infection, PCT is also proposed as a predictor for AKI in various clinical settings [13,14,15,16].

Recently, a meta-analysis conducted by Feng et al. [17] disclosed that PCT might be a helpful predictor for AKI development, and the diagnostic accuracy of PCT for AKI was lower in the septic population than in the population without sepsis. Although this topic was clinically relevant and crucial, the results should be interpreted carefully. Since the study design aimed to enroll studies that provided certain information (namely, true-positive, true-negative, false-positive, and false-negative results) for evaluating PCT’s predictive performance for AKI development, this meta-analysis finally only enrolled 9 studies among the 119 candidate papers. In other words, the authors excluded many relevant studies that could have provided valuable data regarding the AKI-predictive ability of PCT. Additionally, the sensitivities and specificities among the enrolled nine studies had significant heterogeneity [17]. Due to the strict selection criteria and the high heterogeneity among the enrolled studies, the potential bias prohibited the meta-analysis by Feng et al. a representative work to draw a consensus in this field. Clinically, AKI often develops along with infection or inflammation states, which also significantly influence the serum PCT level. The complicated associations among PCT, AKI, and infection/inflammation are essential but not yet clearly addressed.

In the current review, we searched relevant clinical research written in English that evaluated PCT’s predictive ability for AKI in adult patients from four electronic databases (Cochrane Library, EMBASE, MEDLINE, PubMed). The publication period was from the inception until June 2021, and the keywords for the literature search were (“procalcitonin” OR “PCT”) AND (“acute kidney injury” OR “acute renal injury” OR “acute kidney failure” OR “AKI”). For the better understanding of readers, we list some relevant and representative studies in Table 1. Moreover, we also summarize the pathophysiological explanations of PCT elevation in AKI and infection/inflammation states, point out the interference of infection on the AKI-predictive ability of PCT, and recommended directions for further studies to clarify the essential issue.

## 2. PCT and AKI

### 2.1. PCT Could Predict AKI Occurrence

As early as 1975, Ardaillou et al. [36] found that the plasma immunoreactive calcitonin levels were significantly higher in AKI patients, particularly in those in the oliguric phase. Additionally, the immunoreactive calcitonin concentration progressively decreased with time, irrespective of renal function recovery.

PCT’s predictive ability for AKI has been widely studied in cardiovascular patients. From a prospective study enrolling 814 patients with acute coronary syndrome receiving percutaneous coronary intervention, Kurtul et al. [15] found that the patients who developed subsequent contrast-induced AKI had significantly higher serum PCT levels at admission than those who did not. They further demonstrated that admission serum PCT levels independently predicted subsequent contrast-induced AKI, which was defined as a serum creatinine level increase of more than 0.5 mg/dL from baseline within 48 to 72 h following contrast exposure.

In an investigation enrolling 122 cardiac surgical patients by Clementi et al. [16], PCT obtained 48 h after cardiac surgery performed as a stronger predictor for “adverse composite kidney outcomes” than plasma interleukin-6 levels. The “adverse composite kidney outcome” was defined as the occurrence of AKI (by Acute Kidney Injury Network criteria) or worsening of chronic kidney disease (CKD). Similar results from the same study population were shown in the work of Brocca et al. [18].

In addition, Liu et al. [19] evaluated the association between AKI and serum PCT levels using a retrospective study that enrolled 328 patients with acute type A aortic dissection receiving surgeries. This study found that the patients with severe AKI (Kidney Disease: Improving Global Outcomes (KDIGO) stage 3) had statistically higher serum PCT levels than the rest of patients from hospital admission to the seventh postoperative day. However, the differences in serum PCT levels became less significant and only presented on the first postoperative day while comparing the patients with AKI (KDIGO stage 1) and those without stage 1 AKI. Finally, the authors concluded that serum PCT was more accurate in predicting stage 3 AKI than some traditional inflammatory biomarkers, such as C-reactive protein (CRP) and white blood cell count.

The AKI-predictive ability of serum PCT is also disclosed in the population with cerebral vascular illness. Wang et al. [20] conducted a retrospective study to evaluate 214 patients with traumatic brain injury. The multivariate logistic regression analysis demonstrated that serum PCT, age, serum chlorine, and serum creatinine were independent risk factors of AKI. The authors subsequently developed a predictive model using these four risk factors and found that the combined predictive model had significantly higher predictive ability than a single PCT level. Additionally, Schenk et al. [21] retrospectively enrolled 87 critically ill patients with deep-seated intracerebral hemorrhage. They reported that 21 of the 87 patients developed early AKI within the first 48 h of admission, and 9 patients experienced continuous renal replacement therapy during the hospitalization course. The multivariate analysis revealed that “admission PCT level > 0.5 μg/L” was an independent and significant predictor for necessitating continuous renal replacement therapy in these patients with intracerebral bleeding.

As to the critically ill patients, Jia et al. [22] retrospectively included 577 patients admitted to the intensive care unit (ICU) to determine the AKI-predictive ability of some individual or combined risk factors. The study found that urine (tissue inhibitor of metalloproteinase-2 [TIMP-2]) × (Insulin-like growth factor binding protein 7 [IGFBP7]) concentrations were of practical screening value to recognize high-risk patients of moderate to severe AKI (KDIGO stages 2 and 3) within the first seven days of ICU admission. The rest of the independent predictors for severe AKI included serum PCT > 0.5 μg/L at admission, age > 65 years, and CKD. Furthermore, combining these biomarkers and risk factors performed even better for risk assessment of AKI among these critically ill patients.

**Figure 1:**
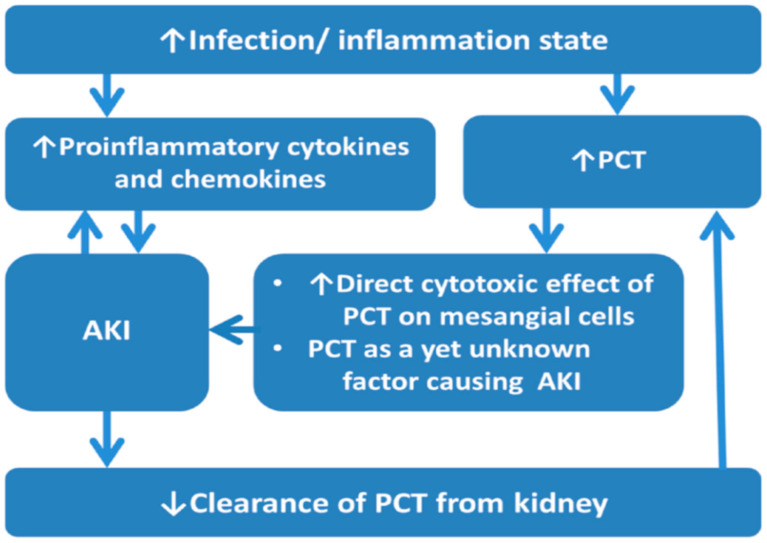
The association between PCT and AKI in infection/inflammation state. Abbreviations: AKI, acute kidney injury; PCT, procalcitonin.

To date, serum PCT level is found higher in AKI patients compared with those without AKI in varied clinical settings. The potential explanations for the association between elevated PCT levels and AKI in infection/inflammation situations include: (1) AKI is associated with elevated proinflammation cytokines and chemokines, such as interleukin-1, interleukin-6, and tumor necrosis factor-α, involved in the infection and inflammation state [37]. (2) Infection and inflammation state increase serum PCT concentration, and kidneys might also contribute to PCT production under this situation [7]. (3) PCT has a direct cytotoxic effect on mesangial cells, causes mesangial cell apoptosis, and plays a yet unknown role in the pathogenesis of AKI [32,38]. (4) The diminished clearance of PCT from the kidney, even in the early stage of AKI before a significant serum creatinine elevation [17], causes elevated PCT concentration and subsequent kidney damage. (Figure 1)

**Figure 2:**
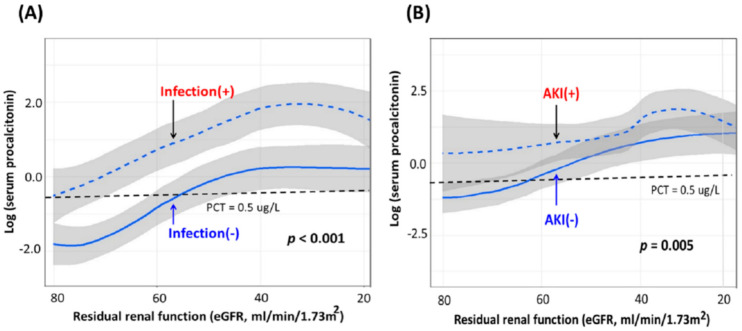
Serum PCT levels between groups stratified by (**A**) infection and (**B**) AKI. Note: The figures were modified from the work of Huang et al. [25]. Abbreviations: AKI, acute kidney injury; eGFR, estimated glomerular filtration rate; PCT, procalcitonin.

Renal function impairment may associate with elevated serum PCT levels [24,39], partially because of the decreased PCT elimination from the kidney [40]. However, the elevated plasma PCT level in the AKI population might not be merely a sequence of impaired renal function as in CKD patients. Our previous work addressed the essential relationship of serum PCT levels with residual renal function, the presence of infection, and AKI [25]. In that study, serum PCT level showed an increasing trend along with the worsening residual renal function (indicated by the decreasing estimated glomerular filtration rate (eGFR)) irrespective of the existence of infection (Figure 2A) and AKI (Figure 2B). Moreover, significantly higher serum PCT concentrations were revealed in infected patients compared with non-infected patients and in AKI patients than in those without AKI at the same corresponding eGFR levels. The above findings indicated that AKI was associated with serum PCT level elevation and that the association was more potent than residual renal function [25] (Figure 2).

The study by Jeeha et al. [32] excluded patients with AKI at admission and measured serum PCT levels before AKI onset. Thus, any rise in PCT levels was less likely to be caused by the decreased excretion from AKI [32]. Additionally, serum PCT levels were significantly associated with some AKI biomarkers, such as neutrophil gelatinase-associated lipocalin (NGAL) and Acute Physiology and Chronic Health Evaluation II scores in infected patients [41]. Since AKI is often associated with inflammation and severity of illness involving hemodynamics and oxygenation [42], this information provides a crucial pathophysiological implication between PCT and AKI.

### 2.2. PCT Could Predict AKI Recovery

In addition to predicting AKI development, PCT was disclosed to have a role in predicting AKI recovery. Itenov et al. [23] conducted a multicenter retrospective study to investigate the role of endothelial damage in the pathogenesis of AKI. This study demonstrated that a higher concentration of PCT and soluble thrombomodulin at admission served as indicators for more severe endothelial damage and predicted a lower probability of AKI recovery.

## 3. PCT, AKI, and Infection/Inflammation

### 3.1. Could PCT Predict AKI in Patients with Infection/Inflammation? For!

A retrospective study including 440 cardiac surgical patients conducted by Heredia-Rodríguez et al. [24] disclosed that the patients with AKI had significantly higher serum PCT levels than those without AKI on a few postoperative days in both patient groups with and without infection. These results demonstrated that the existence of infection did not influence the AKI-predictive ability of PCT.

**Figure 3:**
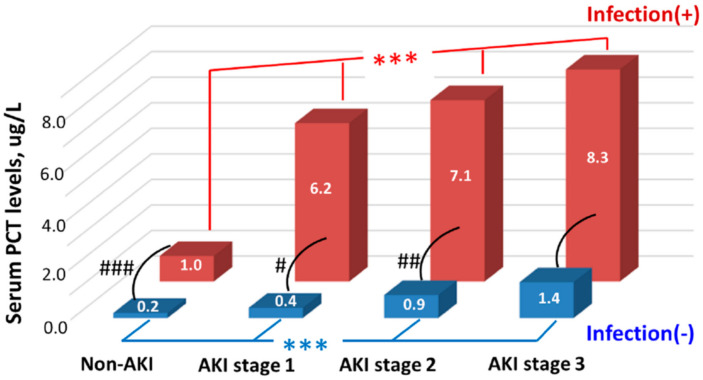
Serum PCT levels among groups stratified by AKI stages and infection states. Note: We used the median values to express the levels. *** indicated *p* < 0.001 among groups with different AKI stages. #, ##, ### indicated *p* < 0.05, <0.01, <0.001, respectively, between infected and non-infected groups. The figure was modified from the work of Huang et al. [25]. Abbreviations: AKI, acute kidney injury; PCT, procalcitonin.

As to the critically ill patients, our team had retrospectively analyzed 330 critically ill patients hospitalized in the ICU to evaluate the complicated associations among serum PCT levels, infection, AKI, and residual renal function [25]. The authors found that serum PCT levels begin to elevate from KDIGO stage 1 AKI. In addition, serum PCT levels increased with worsening AKI severity (indicated by the increasing folds of serum creatinine elevation, from non-AKI to stage 3 AKI) and worsening residual renal function (indicated by the decreasing eGFR). These trends persisted in both infected and non-infected groups, although the infected patients had significantly higher serum PCT concentrations than the non-infected patients. Finally, the authors confirmed that serum PCT level measured within 24 h after ICU admission was an independent predictor of AKI irrespective of infection [25]. Figure 3 clearly shows the serum PCT concentrations in subgroups with various AKI stages and infection states. Although the infected patients had significantly higher serum PCT levels than those without infection in most of the corresponding AKI stages, the serum PCT levels had good differentiation ability among various AKI stages in both infected and non-infected populations [25].

The above findings were consistent with the single-center, retrospective study by Chun et al. 2019 [26] that enrolled 790 critically ill patients, including 266 (33.7%) patients who developed AKI. The AKI patients had a higher percentage of having sepsis than those without AKI. After adjusting for comorbidities, clinical factors, and laboratory results, the serum PCT levels were significantly associated with AKI occurrence. A serum PCT > 0.315 μg/L at admission was an independent risk factor for AKI in both the sepsis and non-sepsis groups.

Several studies focusing on the population with infection or inflammation had also demonstrated the AKI-predictive ability of PCT [13,14,27,28,29,30,31]. For example, Huang et al. [14] included 305 critically ill patients with acute pancreatitis to demonstrate that serum PCT levels at ICU admission were 100-fold higher in AKI patients than those without AKI. Additionally, serum PCT concentrations significantly decreased from the day of AKI to day 28 in survivors, while in non-survivors, the serum PCT levels also increased from AKI onset day to the day of death but did not reach statistical insignificance. The authors further found that PCT’s predictive power for AKI occurrence was significantly superior to serum amyloid A, CRP, and interleukin-6.

Another prospective study by Nie et al. [13] enrolled 1361 patients with clinically suspected infection. The investigation found that a higher serum PCT concentration was positively associated with a higher AKI occurrence rate and stated that PCT could predict AKI patients with infection. Furthermore, PCT performed better in predicting AKI than some well-known infection biomarkers such as CRP and interleukin-6. In addition, serum PCT level was higher in patients with AKI than those without AKI in sepsis patients at the emergency department [27] and was independently correlated with AKI in the population with septic shock [28].

It is worth mentioning the AKI-predictive ability of PCT in patients with the pandemic severe acute respiratory syndrome coronavirus 2 (SARS-CoV-2) since an increasing body of evidence showed the kidney involvement of this virus infection. Hardenberg et al. [29] conducted a multicenter observational cohort study enrolling 223 consecutive patients with SARS-CoV-2 infection to evaluate AKI evolution. The authors reported that 31% of these patients developed severe AKI (KDIGO stage 3), and 95.7% of these severe AKI patients required renal replacement therapy. Serum PCT levels at admission, mechanical ventilation, vasopressor therapy, and white cell count were independent time-varying predictors for severe AKI. A subsequent sensitivity analysis confirmed these results. Several other studies had presented similar results in the population with SARS-CoV-2 infection in either critically ill [30] or less critically ill [31] settings.

### 3.2. Could PCT Predict AKI in Patients with Infection/Inflammation? Against!

Nonetheless, some studies showed conflicting results regarding PCT’s predictive ability for AKI in the infection population. For example, the retrospective study by Jeeha et al. [32] found that serum PCT could not predict AKI in septic patients, although it had optimal predictive power for AKI in non-septic patients. This study enrolled 201 critically ill patients who had no known CKD or AKI history. The study revealed that a higher serum PCT level (≥10 μg/L) compared with a lower PCT level (<10 μg/L) at admission was significantly associated with a higher AKI incidence within seven days of ICU admission (54.8% versus 23.9%, *p* < 0.001). However, the subsequent multivariate analysis reported that a higher serum PCT (≥10 μg/L) could only independently predict subsequent AKI in the non-septic subgroup.

Another retrospective study by Godi et al. [33] verified the utility of combining urinary [TIMP-2] × [IGFBP7] and serum PCT levels at admission for predicting AKI. The cut-off points of serum PCT and [TIMP-2] × [IGFBP7] were set as 0.5 μg/L and 0.3 (ng/mL) 2/10,000. Among the 433 enrolled critically ill patients, 168 (38.8%) had at least KDIGO stage 1 AKI within 48 h of ICU admission. The presence of either biomarker significantly associated with a higher risk of AKI development, while a combination of these two biomarkers demonstrated better predictive ability for AKI occurrence than the two individual biomarkers alone. However, subgroup analysis demonstrated that serum PCT > 0.5 μg/L was only an independent predictor for AKI occurrence in non-sepsis patients but not in the septic population. Consistent with the above studies, other studies did not demonstrate the independently predictive role of serum PCT for AKI in critically ill patients with A/H1N1 virus infection [34] or influenza infection [35].

### 3.3. The Inference of AKI on PCT’s Predictive Ability for Infection

In the current narrative review focusing on the interference of infection on PCT’s predictive ability for AKI, it is also crucial to mention and discuss the possible interference of AKI on the accuracy of PCT’s predictive ability for infection. Infection and inflammation have been reported to have a more potent influence than AKI in association with PCT elevation, and PCT is good at detecting infection. Thus, it is not surprising that PCT could detect infection in AKI patients. However, conflicting findings exist.

Takahashi et al. [41] analyzed 403 blood specimens (229 specimens with infection and 174 specimens without infection) from 91 patients to evaluate the role of plasma PCT for predicting bacterial sepsis under the inference of AKI. A total of 232 specimens were diagnosed with AKI, defined by NGAL ≥ 150 ng/mL. The diagnostic accuracy of plasma PCT for sepsis was even better in AKI patients than in those without AKI, although PCT’s cut-points for diagnosing sepsis were higher in AKI patients. Additionally, in patients after major aortic surgery, Amour et al. [39] found that PCT is a helpful biomarker for detecting bacterial infection. However, the diagnostic accuracy of PCT is not significantly different between the AKI and non-AKI groups with different PCT cut-points for diagnosing infection.

Nevertheless, Nakamura et al. [9] found that serum PCT had an adequate ability for diagnosing sepsis in mild AKI patients (RIFLE-R/I), but the diagnostic accuracy of PCT was significantly blunted with the presence of severe AKI (RIFLE-Failure). Furthermore, Heredia-Rodríguez et al. [24] disclosed that in cardiac surgical patients, AKI patients had significantly higher serum PCT concentrations than non-AKI patients during the ten postoperative days, irrespective of the presence of sepsis. Moreover, among the patients without AKI, the infected patients had significantly higher PCT levels than the non-infected patients, but this significant difference diminished in the AKI group. PCT’s diagnostic value for infection was significantly compromised by the existence of even mild AKI with serum creatinine level ≥ 2.0 mg/dL. These results indicated that the existence of AKI had a significant influence on PCT level and could compromise the diagnostic value of PCT as a biomarker of infection.

## 4. Perspectives and Directions for Further Investigation

An increasing body of evidence supports the role of PCT in predicting AKI occurrence in several clinical settings. Although PCT is not yet listed as a “common AKI biomarker” in the consensus statement of the Acute Disease Quality Initiative (ADQI), the consensus statement recognized the potential of PCT and called for further investigations to evaluate PCT’s role in identifying patients with risk for AKI [43].

According to their characteristics, AKI biomarkers could be categorized into stress biomarkers, damage biomarkers, and functional biomarkers. Functional biomarkers (such as serum creatinine and urine amount) denote “organ failure”, while stress biomarkers and damage biomarkers reflect responses at the cellular level [44]. Furthermore, both functional biomarkers and damage biomarkers play specific roles in evaluating kidney damage. Thus, ADQI consensus suggests subgrading AKI stages 1 to 3 by combining these two biomarkers [43]. Additionally, the ADQI consensus also recommends applying a combination of functional and damage biomarkers, accompanying by clinical presentation, to identify high-risk patients, prompt the diagnostic accuracy of AKI, and improve the quality of care [43].

In the future, further investigations are still warranted to clarify the role and utilization of PCT as an AKI biomarker. Regarding a potential AKI biomarker, we encourage more experimental studies to clarify PCT’s characteristics (such as a stress marker or a damage marker) and the potential roles in clinical practice (such as for AKI assessment, prediction AKI occurrence or recovery, AKI diagnosis, or AKI severity grading) [43]. It is also essential to determine the anatomical location of the part of the kidney that is associated with elevated PCT levels, although existing data reported that PCT causes mesangial cell apoptosis [38]. Localizing injury anatomy provides a rational framework for interpreting the molecular responses to various environmental stimuli [44].

Regarding the application of PCT, several potential strategies could be utilized. For example, investigators could use “individual PCT cut-points for individual population”, “the change (in percentage or values) of PCT levels within a period”, and “a single versus serial measurements of PCT” to evaluate the association among PCT, AKI, and infection. Additionally, PCT, other functional or damage biomarkers, and clinical assessment could be used in combination or alone to evaluate or compare the accuracy of risk assessment, AKI prediction, AKI diagnosis, prognosis prediction, or cost-effectiveness of care [10,23,43,45,46,47,48].

After the AKI-predictive ability of PCT is determined, physicians could use it to identify earlier those patients with a higher risk of AKI occurrence and to pay more attention to avoiding nephrotoxic medications or procedures during hospitalization. Hopefully, the utilization of this biomarker could improve the prognoses of patients at high risk of developing AKI.

## 5. Conclusions

According to the increasing body of evidence, serum PCT level is a potential biomarker for predicting AKI in many clinical settings, including those patients with infection. Nevertheless, further investigations are warranted to confirm PCT’s predictive ability for AKI and the inference of infection and to extend PCT utilization to improve the prognoses of patients at high risk for AKI.

## Figures and Tables

**Table 1:** Studies evaluating PCT’s predictive ability for AKI and the inference of infection/inflammation.

Categories	Populations	Main Results	Type of Study	Reference
**Studies supporting PCT as a predictor of AKI occurrence and recovery in patients without specifying infection/inflammation states**				
	Patients with acute coronary syndromes received urgent percutaneous coronary intervention (*n* = 814)	Serum PCT levels (at admission) are independently associated with the risk of subsequent contrast-induced AKI.	Single-center, prospective study	Kurtul A et al. 2015 [15]
	Cardiac surgical patients (*n* = 122)	Serum PCT levels (48 h after surgery) showed a more substantial predictive value for adverse kidney outcomes, including AKI, than IL-6.	Single-center, prospective study	Clementi A et al. 2017 [16]
	Cardiac surgical patients (*n* = 122)	Serum PCT levels (48 h after surgery) had an excellent predictive value of cardiac surgery-associated AKI.	Single-center, prospective study	Brocca A et al. 2017 [18]
	Surgical patients with acute type A aortic dissection received surgery (*n* = 328)	Serum PCT levels were significantly different between patients with different AKI stages. Serum PCT serves as a valuable biomarker in predicting severe AKI. (KDIGO stage 3)	Single-center, retrospective study	Liu et al. 2020 [19]
	Patients with traumatic brain injury (*n* = 214)	Serum PCT level was an independent risk factor of AKI.	Single-center, retrospective study	Wang et al. 2020 [20]
	Critically ill patients with spontaneous deep-seated intracerebral hemorrhage (*n* = 87)	Serum PCT level > 0.5 μg/L at admission was an independent and significant predictor for AKI needing continuous renal replacement therapy.	Single-center, retrospective study	Schenk et al.2021 [21]
	Critically ill patients admitted to ICU (*n* = 577)	Serum PCT level > 0.5 μg/L at admission was an independent predictor for AKI (KDIGO stage 2 and 3) within seven days following ICU admission.	Single-center, retrospective study	Jia, et al. 2020 [22]
	Critically ill patients with AKI (*n* = 213)	Higher serum PCT level at admission was associated with a lower chance of renal function recovery among AKI patients.	Multicenter, retrospective study	Itenov et al. 2017 [23]
**Studies supporting PCT’s predictive ability for AKI in patients with infection/inflammation**				
	Cardiac surgical patients with severe sepsis, septic shock, or SIRS (*n* = 440)	Serum PCT levels were significantly higher in AKI patients than those without AKI during the ten postoperative days, irrespective of sepsis.	Single-center case-control study	Heredia-Rodriguez et al. 2016 [24]
	Critically ill patients (*n* = 330)	Serum PCT level at ICU admission was an independent predictor of AKI in both infected and non-infected patients.	Single-center, retrospective study	Huang et al. 2020 [25]
	Critically ill patients (*n* = 790)	Serum PCT level (as a continuous variable or a categorical variable with PCT > 0.315 μg/L) at admission was an independent risk factor for AKI, regardless of the existence of sepsis.	Single-center, retrospective study	Chun et al. 2019 [26]
	Critically ill patients with acute pancreatitis (*n* = 305)	Serum PCT levels at ICU admission predicted AKI and prognosis.	Single-center prospective study	Huang et al. 2013 [14]
	Mixed hospitalized medical/surgical patients with infection (*n* = 1361)	Serum PCT was a useful predictive biomarker for AKI.	Single-center prospective study	Nie et al. 2013 [13].
	Sepsis patients in the emergency department (*n* = 140)	Serum PCT levels were higher in AKI patients than in those without AKI.	Single-center, retrospective study	Park et al. 2019 [27]
	Patients with bacterial septic shock (*n* = 157)	Serum PCT level was independently associated with septic shock-induced AKI.	Single-center retrospective study	Fu et al. 2021 [28]
	Patients with SARS-CoV-2 infection (*n* = 223)	Serum PCT level at admission was an independent time-varying risk factor of AKI (KDIQO stage 2 and 3).	Multicenter observational study	Hardenberg et al. 2021 [29]
	Critically ill patients with SARS-CoV-2 infection (*n* = 42)	A higher serum PCT level at ICU admission was associated with AKI occurrence.	Single-center observational study	Barragan et al. 2021 [30]
	Patients with SARS-CoV-2 infection (*n* = 116)	Serum PCT level > 0.1 ng/mL was an independent factor for AKI.	Single-center, retrospective cohort study	Wang et al. 2020 [31]
**Studies not supporting PCT’s predictive ability for AKI in patients with infection/inflammation**				
	Critically ill patients (*n* = 201)	Serum PCT level at admission was an independent predictor for AKI (within seven days after admission) in non-septic patients but not in septic patients.	Retrospective observational study	Jeeha et al. 2018 [32]
	Critically ill patients (*n* = 433)	Serum PCT level > 0.5 μg/L was an independent predictor for AKI occurrence in non-sepsis patients but not in the septic population.	Single-center, retrospective study	Godi et al. 2020 [33]
	Critically ill patients with A/H1N1 virus infection and ARDS (*n* = 32)	Serum PCT level was not an independent predictor for AKI in ARDS patients associated with A/H1N1 infection.	Retrospective study	Cruz-Lagunas et al. 2014 [34]
	Critically ill patients with influenza infection but no bacterial co-infection (*n* = 663)	Serum PCT level was not a predictor for AKI and could hint at a potential bacterial infection.	Secondary analysis of a prospective multicenter study	Rodríguez et al. 2018 [35]

Abbreviations: ARDS, acute respiratory distress syndrome; AKI, acute kidney injury; ICU, intensive care unit; IL-6, interleukin 6; KDIQO, Kidney Disease: Improving Global Outcomes; PCT, procalcitonin; SARS-CoV-2, severe acute respiratory syndrome coronavirus 2; SIRS, systemic inflammatory response syndrome.

## Data Availability

Not applicable.
